# Contrasting spatial, temporal and environmental patterns in observation and specimen based species occurrence data

**DOI:** 10.1371/journal.pone.0196417

**Published:** 2018-04-26

**Authors:** James D. M. Speed, Mika Bendiksby, Anders G. Finstad, Kristian Hassel, Anders L. Kolstad, Tommy Prestø

**Affiliations:** Department of Natural History, NTNU University Museum, Norwegian University of Science and Technology, Trondheim, Norway; State Museum of Natural History, GERMANY

## Abstract

Species occurrence data records the location and time of an encounter with a species, and is valuable for many aspects of ecological and evolutionary analyses. A key distinction within species occurrence data is between (1) collected and preserved specimens that can be taxonomically validated (i.e., natural history collections), and (2) observations, which are more error prone but richer in terms of number and spread of observations. In this study we analyse the distribution in temporal, spatial, taxonomic and environmental coverage of specimen- and observation based species occurrence data for land plants in Norway, a region with strong climatic and human population density gradients. Of 4.8 million species occurrence records, the majority (78%) were observations. However, there was a greater species richness in the specimen record (N = 4691) than in the observation record (N = 3193) and most species were recorded more as specimens than observations. Specimen data was on average older, and collected later during the year. Both record types were highly influenced by a small number of prolific contributors. The species most highly represented in the observation data set were widespread or invasive, while in the specimen records, taxonomically challenging species were overrepresented. Species occurrence records were unevenly spatially distributed. Both specimen and observation records were concentrated in regions of Norway with high human population density and with high temperatures and precipitation, but in different regions within Norway. Observation and specimen records thus differ in taxonomic, temporal, spatial and environmental coverage for a well-sampled group and study region, potentially influencing the ecological inferences made from studies utilizing species occurrence data. The distribution of observation data dominates the dataset, so inferences of species diversity and distributions do not correspond to the evolutionary or physiological knowledge of species, which is based on specimen data. We make recommendations for users of biodiversity data, and collectors to better exploit the complementary strengths of these distinct biodiversity data types.

## Introduction

Recent years have seen a huge expansion in the quantity of georeferenced species occurrence data worldwide [1]. The sources of such data commonly are citizen science observations, and digitized natural history collection data. During the current global biodiversity crisis this data has proved to be a valuable source of information regarding species distributions and habitat associations, responses to climatic changes (e.g. [2, 3]), and conservation efforts (e.g. [4, 5]). However, species occurrence data is subject to several weaknesses. First, there are biases in geographical coverage of species records, taxonomic biases and temporal biases (e.g. [6, 7]). Secondly, the lack of information about the observation process often makes it often impossible to draw inference on detection probability and false absences, hence reliable inferences regarding species distributions and their relation to environmental drivers cannot readily be made [8]. Thirdly, species occurrence data may also be subject to direct errors (e.g. [9, 10]), despite ongoing efforts to correct these [11]. Errors or inaccuracies may occur in the geo-referencing (i.e. the occurrence was not actually present at the location indicated), temporal reference (error or lack of specificity in the date given to the record), or taxonomic error (a misidentified taxon, or an outdated identification due to taxonomic revisions). Although such errors are not widespread [12], the ecological inferences drawn from these data may be substantially affected [7, 13, 14]. Within a relatively short timeframe, international initiatives for ecological data availability (e.g. the Global Biodiversity Information Facility, GBIF) have created opportunities for the use of large quantities of species occurrence data. While this opens new doors for research, it also makes it paramount that we understand the limitations in the data quality and make our best efforts to correct for potential biases in the data.

There has been considerable discussion and research into differences in biases and errors between citizen science data and professionally collected species occurrence data (e.g. [15, 16]). However, much of this discussion does not acknowledge there is a distinction between observation and specimen (or sample) based occurrence records, which is crucial for aggregated species occurrence databases. Both specimen and observation datasets may be comprised of a mixture of professional or citizen science collections and structured or unstructured sampling [17]. However, when the occurrence of a species is documented with a preserved specimen in natural history collections, the taxonomic classification is reproducible and traceable. Here, the physical sample can be re-examined, thereby allowing the taxonomic classification of these occurrences to be validated and updated following taxonomic revisions. In contrast, observation-only data, collected by trained natural historians or by citizen scientists alike, lacks physical specimens to back-up the record [18]. While photographs can be used to document observations, for many taxa, a sample is required to reliably identify a species for example using microscopy [19]. There are therefore concerns regarding the certainty and therefore utility of observation based species occurrence data [20, 21].

The utility of specimen data for macroecological research is limited due to spatial and taxonomic gaps [21]. Meanwhile, the quantity of observation-based species occurrence data has rapidly increased over the past few years [1]. Observation data has the advantage of lower required effort, and does not require the mortality or disturbance to organisms caused by collection of specimens [22]. In many datasets it is therefore comprises a higher number of biodiversity records. Specimen data has the advantage of taxonomic transparency. In addition, natural history collections preserve specimens that can subsequently be used for georeferencing studies of genetics or evolution (e.g. [23, 24]), phylogeography [25], physiology (e.g. [26]) or phenology [27]. Yet, biases and low sample sizes within natural history collections have constrained the application of specimen data in some contexts [28, 29], often reflecting the geographic and taxonomic preferences of a small number of contributors to such collections. Some of these limitations could be resolved through developing links with observation data that has the advantage of a potentially far greater sampling effort, and wider geographical reach. Specimen-based species occurrence records can provide an up-to-date taxonomic reference that can be used to validate observation based species occurrence data, in addition to preserved samples for future genetic or physiological analyses. A prerequisite to this is to first understand how such datasets differ in time, space and quality.

In this study, we set out with the objective of testing the concordance between specimen- and observation-based species occurrence data. We compare temporal, taxonomic, spatial and environmental distributions between these two record types using a dataset of well-sampled taxa in a well-sampled region, namely land plants in Norway. We test the hypotheses that observations are in **temporal** terms (H1) more recent than specimen records [1]. In **taxonomic** terms, we test the hypotheses that observations (H2) record a lower diversity of plants in terms of species richness and evenness [30], but (H3) are more closely related to taxonomic prevalence (i.e. more common species are recorded more frequently) and (H4) sampled by a larger number of recorders than specimen records [18]. In **spatial** terms, we hypothesise that observations are (H5) more geographically widespread than preserved specimens. Finally, in **environmental** terms, we hypothesise that the observations are (H6) more prevalent than specimen records in warmer and wetter parts of Norway with higher human population densities [18]. The results of these analyses will be used to suggest steps to maximise synergies between biodiversity data types.

## Material and methods

The scope of this study was limited to all land plant (Embryophyta) occurrence records within Norway. This taxonomic scope was selected since it is a well-recorded taxon spanning a range of phyla varying in difficulty of field identification. The geographical scope incorporates a well-surveyed region with strong gradients in human population density and climatic conditions. All georeferenced species-occurrence data with no known spatial issues within the kingdom Plantae and the country Norway was downloaded from GBIF on 6^th^ October 2017 [31]. This dataset included 5 308 907 occurrences. The GBIF backbone taxonomy was used within this manuscript [32].

This dataset was quality controlled by undertaking the following steps. First, records made during 2017 were removed, since there may be a lag in digitising specimen data. Next, Plantae taxa outside the sub-kingdom of Embryophyta (i.e. algae *s*.*l*.) were excluded, as were records from the phylum Anthocerotophyta (hornworts) due to very few records (176) from only the far south of Norway. Occurrences of Bryophyta (mosses), Marchantiophyta (liverworts) and Tracheophyta (vascular plants) were retained. The majority of records were human observations or preserved specimens, other record types together accounted for 1.2% of records (mostly ‘Unknown basis of record’), and were excluded from the final data set. Records with missing data for species were excluded, as were duplicate records (records with the same species, date, basis of record, recorder and coordinates). Finally, records that did not fall within 1 km of terrestrial Norway (GADM, i.e. not including Svalbard and Jan Mayen) were also excluded. This removed any records that were erroneously located at sea. The final data set included 4 763 810 species occurrence records. No further filters were applied to the data and the dataset was used ‘as is’. Data processing and analyses were carried out in the R statistical environment [33], running on a Linux installation of R Studio Server.

### Temporal distribution

We tested for differential temporal coverage of the observation and specimen occurrence records both through time and within years. We used a Mann-Whitney two-sample U test with year of occurrence as the dependent variable (due to non-normality), and a t test with Julian day of occurrence (since date of record approximated a normal distribution), to respectively test the null hypotheses that the distribution of year of occurrence and the distribution of date of recording of the different record basis did not differ.

### Taxonomic and recorder distribution

Taxonomic biases between the two record types were investigated at the phylum level and species level. At the phylum level we used a Chi squared test to investigate whether each phylum was represented by each record type as expected given the total number of occurrences within each record type (i.e. the expectation that specimens and observations are equivalently represented). To test for differences in species richness, we again used a Chi-squared test to investigate whether the number of species observed per record type and phylum differed from expectations. We also analysed the ratio of observation to specimen data in species split by taxonomic class. Species rank-abundance curves for each record type were plotted to visualise species dominance within each record type [34]. Recorder rank-abundance curves were also plotted, counting the number of species occurrence records made by each unique recorder (as provided in the dataset; differences in formatting of names or multiple co-recorders were treated as unique recorders).

A null model would expect that records of species occurrences would be proportional to the commonness of each species. Commonness could be assessed in terms of the total population size (or biomass or cover) of a species, or how widespread a species is [35]. To investigate whether recorder effort was related to species prevalence and identify over or under represented species in each record basis, the number of occurrences per species and record type was plotted against the geographic range of that species assessed by the number of 10 × 10 km^2^ grid cells that a species was recorded within. Species highly over- or under-represented species in each record type were identified as those with the greatest absolute residuals from a quadratic regression between number of occurrences and geographic range.

### Spatial distribution

We investigated spatial biases in the species occurrence records of Embryophyta within Norway by first counting the number of occurrences within 10 x 10 km grid cells across the whole of the country. Probability density functions were plotted from this data for the total number of records in each of the record types, and also by phylum. Next, two-dimensional probability density functions were mapped over mainland Norway to visualise regions where the occurrence of different records was most concentrated. This was achieved using a common approach of a two-dimensional kernel density function, evaluated across a square grid, again across all occurrences within each record type and phylum (e.g. [36]).

### Environmental distribution

To visualise differences in environmental space we used WorldClim bioclimatic data [37]. This is a set of 19 variables derived from monthly temperatures and precipitations to produce more ecologically relevant variables. We used the three bioclimatic variables that were closely associated with the first three axes in a principle component dimensionality reduction exercise; the three axes respectively represented 61.7, 19.9 and 7.4% of the total variation in the data set–a total of 89% [38]. The variables associated with these axes are annual precipitation, mean temperature of the warmest quarter (referred to as mean summer temperature) and the precipitation seasonality (the coefficient of variation of monthly precipitation). In addition, human population density across Norway was taken from the Gridded Population of the World dataset [39] These four variables were resampled using the nearest neighbour method and projected onto a 1 km UTM grid (from a 30 arc second resolution at equator). Values were extracted at each occurrence locality and probability density functions created for each record type as well as across the whole of Norway. A Mann-Whitney two-sample U test was used to test whether the environment where records were made differed between record bases.

The GBIF data used in this study is available here http://dx.doi.org/10.15468/dl.f2guqo

## Results

### Temporal distribution

**Fig 1:**
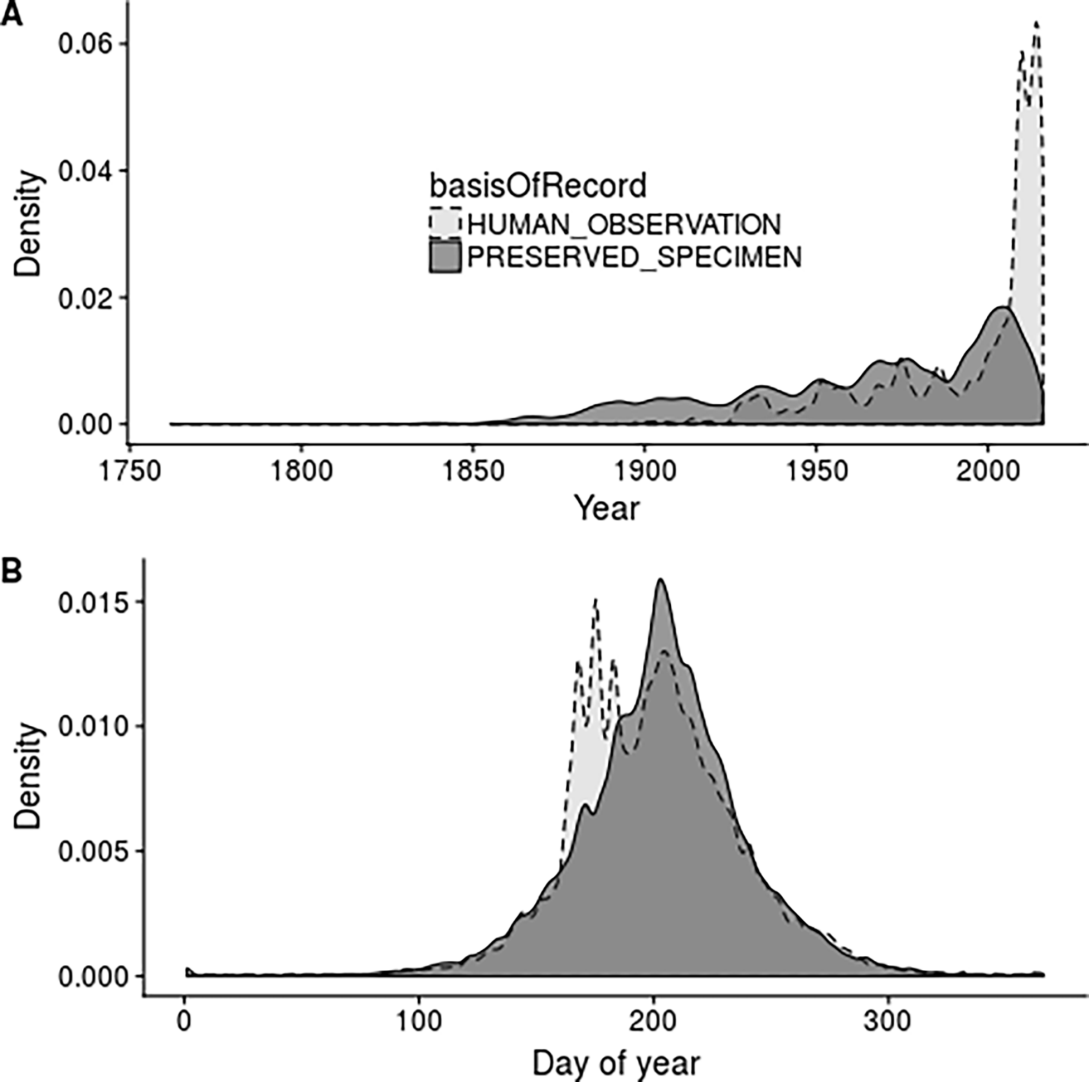
Density plots of (A) year and (B) day of year of plant occurrence records within Norway for observations and specimens.

Preserved specimens and human observation made up 22.4% and 77.6% of the occurrence records in our dataset, respectively. The majority of the human observation records dated from the 21^st^ Century (median 2008, interquartile range 1979–2013) whereas preserved specimens were more evenly distributed over time (median 1974, interquartile range 1938–2000, Fig 1A). This difference was statistically significant (W = 2.97 x 10^12^, P< 0.001). The mean day-of-year of occurrence records was around three days earlier in the case of human observation records (200 i.e. 19^th^ July in a non-leap year, sd = 38) than preserved specimens (203, sd = 36), again a significant difference (Fig 1B, t = 78, df = 1714700, P<0.001).

### Taxonomic and recorder distribution

Tracheophyta (i.e. vascular plants) made up most of the recorded occurrences across both record types (Table 1A). The number of occurrences for each phylum differed significantly depending on the record type (χ^2^ = 309090, df = 2, P<0.001): Tracheophyta were recorded as human observations more than expected, while Bryophyta and Marchantiophyta were recorded as preserved specimens more than expected (Table 1A). The number of species occurrences per phyla did not differ from expectations between record types (Table 1B, χ^2^ = 4.75, df = 2, P = 0.09).

**Table 1:** A. The number of occurrence records by phylum within each phylum and basis of record. The proportion of the total number of division occurrence records by basis of record is shown in parentheses. B. The number of species recorded by each basis of record within each division. The proportion of the total number of species recorded by each record type shown in parentheses.

	A. Recorded occurrences			B. Recorded species		
Phylum	Human observation	Preserved specimen	Total	Human observation	Preserved specimen	Total
Bryophyta	56939 (0.30)	130689 (0.70)	187628	N = 710 (0.73)	N = 964 (0.997)	N = 967
Marchantiophyta	22520 (0.38)	36621 (0.62)	59141	N = 231 (0.74)	N = 313 (1.00)	N = 313
Tracheophyta	3615109 (0.80)	901932 (0.20)	4517041	N = 2252 (0.65)	N = 3414 (0.98)	N = 3485
**Total**	3694568 (0.78)	1069242 (0.22)	4763810	N = 3193 (0.67)	N = 4691 (0.98)	N = 4765

**Fig 2:**
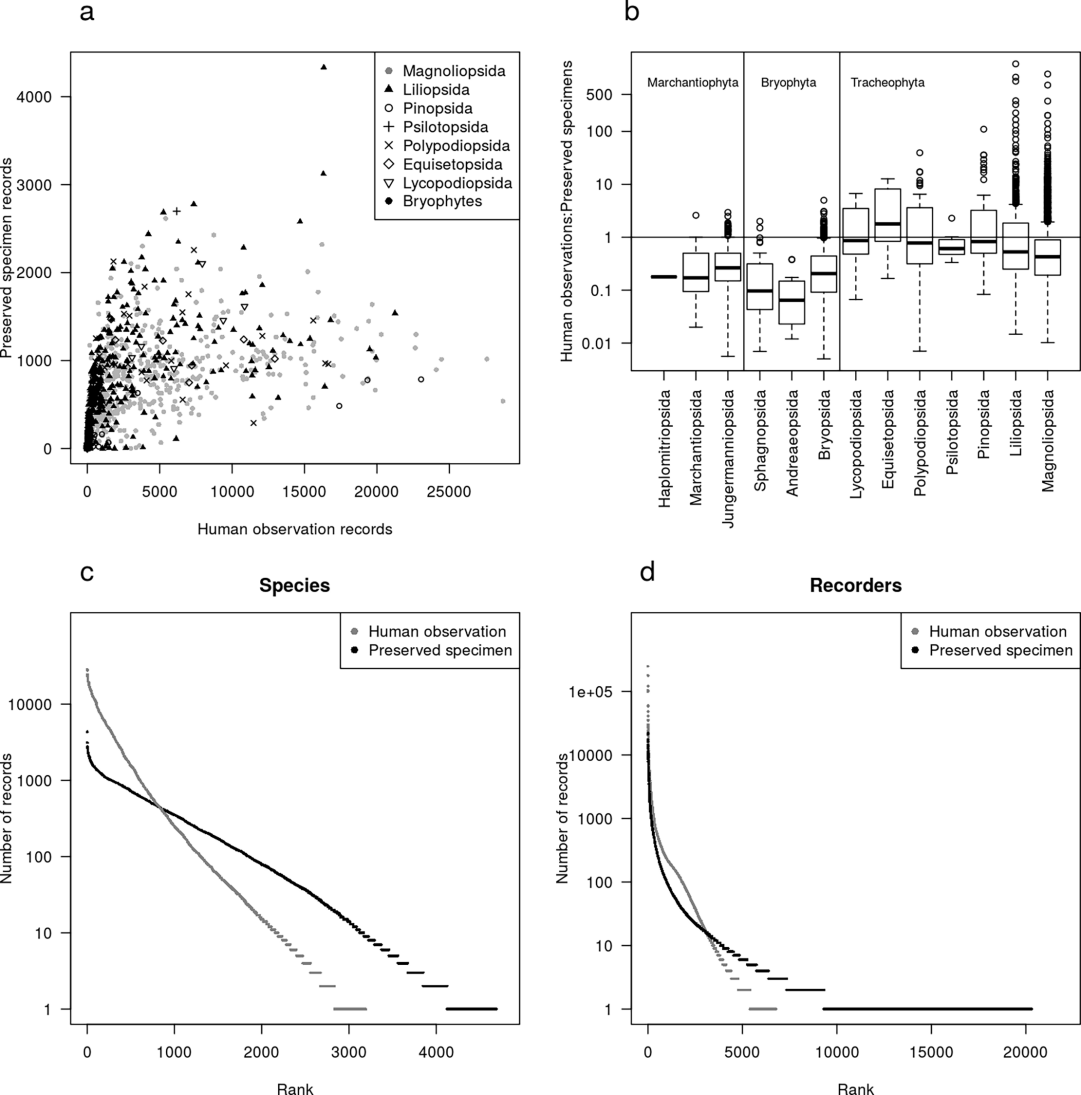
(a) Number of human observation and preserved specimen records of each species. Species are plotted with symbols denoting their taxonomic class (Bryophyta and Marchantiophyta grouped as bryophytes). (b) The ratio of number of observations to number of specimen records for each species grouped by taxonomic class. Boxes represent interquartile ranges, with whiskers 1.5 times this range, and points showing species outside the range. The median is shown by a black line. Values above one show species with more observation records than specimen records. Vertical lines divide classes within the three phyla. (c) Species rank-abundance plot for each record type in the Norwegian Embryophyta dataset, showing the number of records (note log_10_ y-axis) for each species plotted against the species’ rank when ordered from most to least abundant within each record type. Table 2 gives the identity of the 10 most abundant species per record basis. (d) Recorder rank-abundance plot for each record type showing the log number of records made by each recorder, plotted against the recorders’ rank when ordered from the most to least abundant within each record type. S1 Table gives the identity of the 10 most abundant recorders per record basis.

Across all phyla, 4 691 species were recorded as preserved specimens, with 3 193 as human observations (of a total of 4765 unique species; Table 1B). There was a positive correlation between number of records of human observations and preserved specimens (Spearman rank correlation, r = 0.88, P<0.001), but the most sampled species by one record type did not correspond to the most sampled species in the other record type (Fig 2A, Table 2). In all classes of plants, most species were more represented as specimens than observations, with the exception of Equisetopsida, within which most species were better represented as observations than specimens (Fig 2B). However, there was a tendency for bryophyte species to be better represented as specimens than vascular plants (Fig 2B). *Lupinus polyphyllus* was the most recorded species in the human observation record and overall, whilst *Luzula multiflora* was the most abundant species in the preserved specimen record (Table 2). The most recorded species by human observation included many shrub and tree species from the classes Magnoliopsida and Pinopsida, while the most recorded species within preserved specimens were mostly graminoids in Liliopsida (Table 2, Fig 2A, Fig 2B). The human observation record was to a higher degree influenced by a large number of observations of relatively few common species, as compared to the preserved specimen record which had a longer tail (Fig 2C).

**Table 2:** The number of occurrence records of the 10 most abundantly recorded species by each record type. The proportion of the total number of occurrences within each record type represented by each of these species is shown in parentheses. The growth form of the species is shown, as is the range, calculated as the number of 10 × 10 km cells in which the species was recorded as any record type.

Rank	Human observation	Growth form	Range (km^2^)	Occurrences (proportion)	Preserved specimen	Growth form	Range (km^2^)	Occurrences (proportion)
1	*Lupinus polyphyllus*	Herb	139 700	28700 (0.008)	*Luzula multiflora*	Graminoid	287 800	4334 (0.004)
2	*Vaccinium myrtillus*	Dwarf shrub	278 300	27590 (0.007)	*Carex nigra*	Graminoid	276 500	3123 (0.003)
3	*Betula pubescens*	Tree	267 500	24453 (0.007)	*Carex flava*	Graminoid	192 100	2779 (0.003)
4	*Sorbus aucuparia*	Tree	257 100	24095 (0.007)	*Botrychium lunaria*	Fern	173 000	2698 (0.003)
5	*Juniperus communis*	Tree/Tall shrub	272 100	23046 (0.006)	*Carex capillaris*	Graminoid	186 600	2686 (0.003)
6	*Potentilla erecta*	Herb	250 300	22695 (0.006)	*Pyrola rotundifolia*	Herb	153 600	2615 (0.002)
7	*Vaccinium vitis-idaea*	Dwarf shrub	278 300	22642 (0.006)	*Festuca rubra*	Graminoid	261 900	2579 (0.002)
8	*Deschampsia cespitosa*	Graminoid	283 700	21243 (0.006)	*Luzula sudetica*	Graminoid	170 300	2440 (0.002)
9	*Calluna vulgaris*	Dwarf shrub	253 800	20293 (0.005)	*Viola canina*	Herb	199 400	2427 (0.002)
10	*Filipendula ulmaria*	Herb	246 700	19963 (0.005)	*Poa alpina*	Graminoid	182 400	2347 (0.002)

The species occurrence records, based on both record types, were also highly influenced by a few prevalent recorders (Fig 2D). There were more unique recorders in the preserved specimen record (20 307) than the human observation record (6 783). This difference was driven mostly by a large number of recorders of preserved specimens with only one record (Fig 2D). The most prevalent recorder of human observations contributed almost 7% of all human observations and over 5% of the total data set, while the most prevalent contributor of preserved specimen records made 21 906 deposits, or 2% of the herbarium specimens and 0.5% of the total data set (S1 Table).

**Fig 3:**
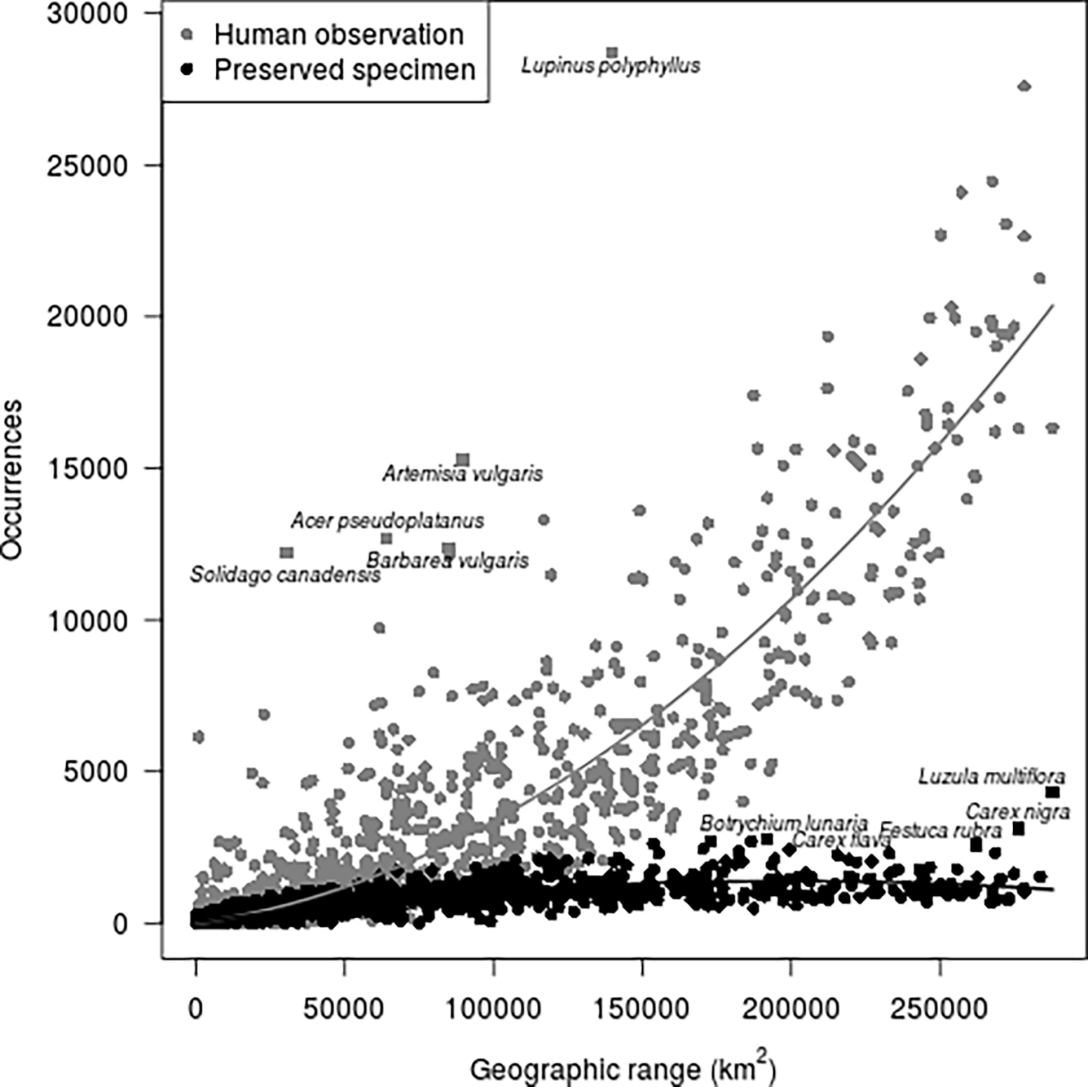
The number of species occurrence records of each record type plotted against the geographic range, here estimated as the number of 10x10 km grid cells within which that species had been recorded (as any record type). Outlying species are plotted as square points and labelled.

There was a positive relationship between geographic range and number of observations (Fig 3). This was steeper for human observations than preserved specimens, and there was more variation around the relationship in the human observations than preserved specimens. Several outliers within the relationship were apparent–that is species that were observed at a greater frequency than their range would otherwise suggest. For the human observation records, these included *Lupinus polyphyllus*, (also the most recorded species overall, Table 2), along with *Artemisia vulgaris*, *Acer pseudoplatanus*, *Barbarea vulgaris* and *Solidago canadensis*. For the preserved specimen records these were all among the ten most sampled species in the specimen record (Table 2).

### Spatial distribution

Spatial bias was assessed by examining the density of species occurrences per 10 x 10 km grid cell. There was no difference in spatial bias for the Tracheophyta (S1 Fig). However, there were significant differences for Bryophyta (W = 1844000, P<0.001, S1 Fig) and Marchantiophyta (W = 1044200, P<0.001, S1 Fig). For both Bryophyta and Marchantiophyta, the human observation records showed more spatial clumping than the preserved specimen record (i.e., the records were more concentrated in fewer cells in the case of human observation records, than preserved specimen records).

**Fig 4:**
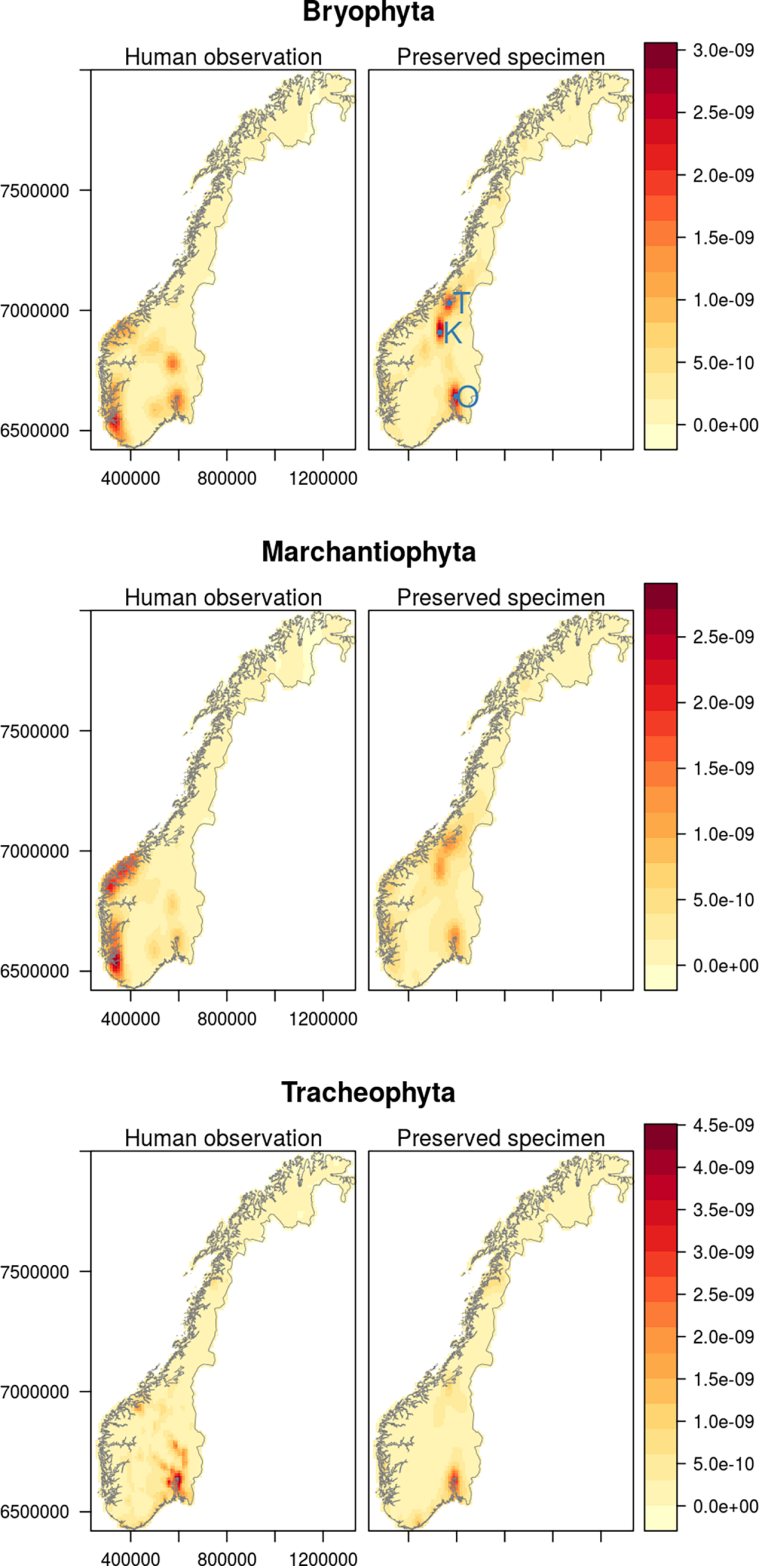
Two dimensional kernel densities showing the probability distribution function (expressed as a percentage) of species occurrences within phyla and record types. Darker shades denote a higher probability of a given occurrence being at that location. Note that the colour scales differ between phyla, but are the same within phyla. Locations referred to in the text are labelled in top right panel; Trondheim (T), Kongsvoll (K) and Oslo (O).

Kernel density maps of geography of spatial bias show high concentrations of both human observations and preserved specimens of Bryophyta and Tracheophyta around Oslo, the Norwegian capital city and most populated region (Fig 4). There are secondary regions of high occurrences for preserved specimens of Bryophyta and Marchantiophyta around the city of Trondheim and the field station at Kongsvoll. However, there were high concentrations of human observations of Bryophyta and Marchantiophyta along the west coast of Norway that were not reflected in the preserved specimen records. Within each of the three phyla, there were no strong correlations between the number of records from either observation-only or preserved specimen based occurrences. The maximum Spearman rank correlation coefficient between human observations and preserved specimens was r = 0.57 for Tracheophyta (S2 Fig). There were stronger correlations within record types between Bryophyta and Marchantiophyta (r = 0.80 and r = 0.71 for human observations and preserved specimens, respectively) and a weaker correlation between Tracheophyta and Bryophyta in preserved specimens (r = 0.41).

### Environmental distribution

**Fig 5:**
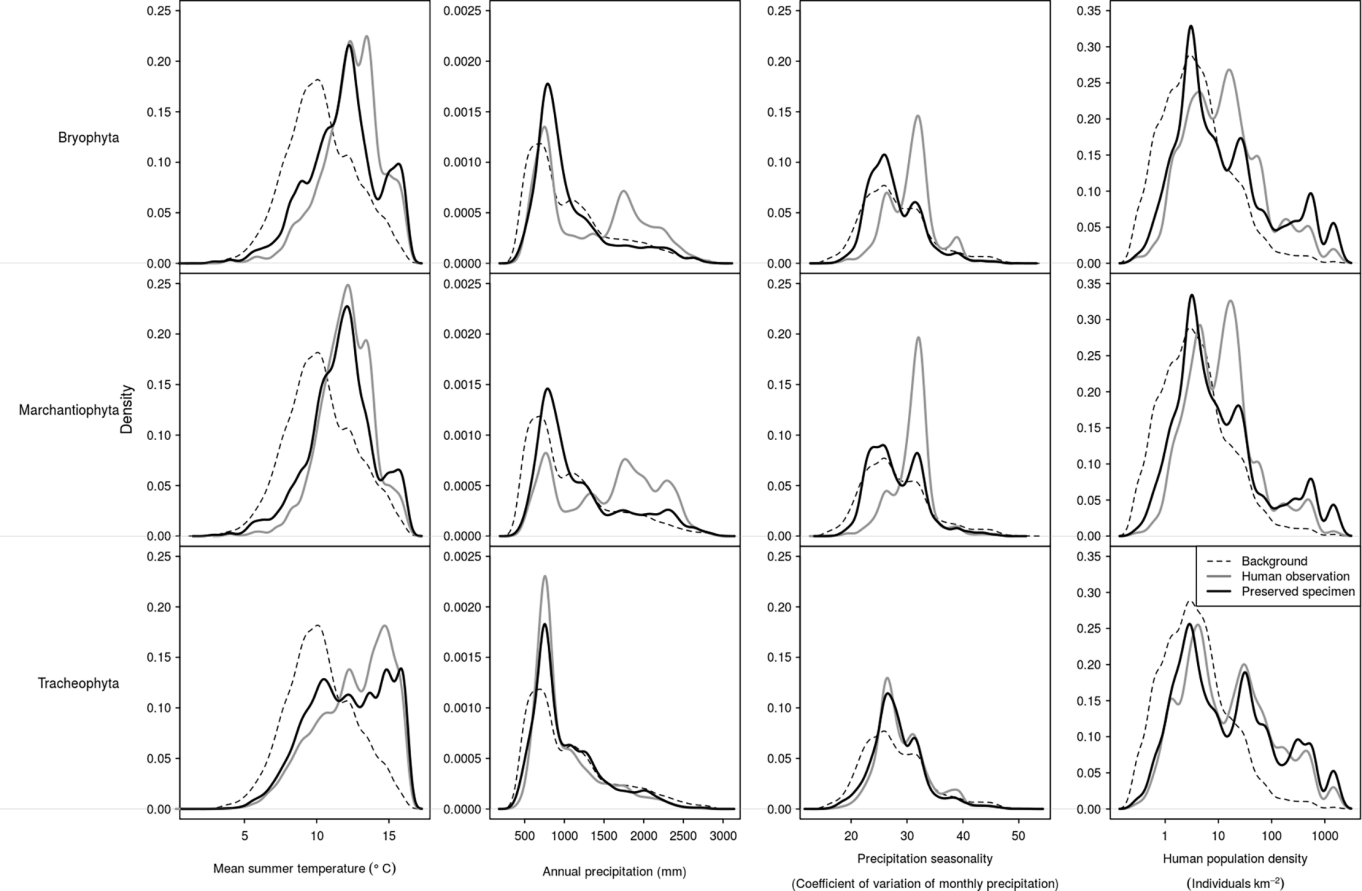
Density plot showing distributions of species occurrence data by record type along the three dominant climatic variables and human population density across Norway. The dashed lines show the total climatic or human population density space within Norway. Rows denote different phyla, while columns show different variables.

The distribution of the species occurrence data according to the three main axes of bioclimatic variation in Norway, along with human population density, is shown in Fig 5. Both human observations and preserved specimens were more commonly recorded in regions that were warmer than Norway as a whole. This was the case for all three phyla. In the case of human observations of Marchantiophyta and Bryophyta, species occurrence records were from regions disproportionally wetter than the whole of Norway. Human observations of these phyla were also made in environments with more seasonal patterns of precipitation than Norway as a whole (Fig 5). The distribution of preserved specimens of Marchantiophyta and Bryophyta, as well as both record types of Tracheophyta, were more similar in terms of precipitation to Norway as a whole (Fig 5). The distribution of species occurrence records significantly differed between human observation and preserved specimens in terms of distribution along all three main bioclimatic axes (Mann Whitney U test, P< 0.001 for all). Both human observations and preserved specimens were sampled from regions of Norway with higher human population density than expected (Fig 5). This difference was similar for Tracheophyta, but for both Bryophyta and Marchantiophyta, the human observation record was more strongly biased in favour of regions with higher human population density.

## Discussion

Here we have shown that the observation and specimen records for Norwegian land plants greatly differ in terms of their distribution in time, taxonomic coverage, space and environment. Such differential distributions have implications for the use of biodiversity data that comprises from species occurrences either recorded as observations, or as specimens preserved in natural history collections. The increasing availability of biodiversity data [6] has enabled many advances in ecological research [40–42]. However, our findings demonstrate that different sources of biodiversity data have distinct patterns in space, time and environment, along with taxonomic coverage, with implications for its application within ecological research.

The digitization of natural history collections [42, 43] and publication of both citizen science and professional species observation data [18, 44] have together driven increased availability of biodiversity data. Recent research has contrasted data distribution and quality collected by citizen scientists and professional ecologists and natural historians, finding different geographic distributions but similar errors and biases (e.g. [15, 16, 45]). Clear taxonomic, temporal and spatial biases are known to exist both in citizen science species occurrence data [46], and in natural history collections [28, 29, 47]. These biases can limit the utility of both preserved specimens and species occurrence data as resources in conservation [48] and certain aspects of research, for example population studies and habitat affinities [28]. However, there is potential combine the benefits of observation and specimen data. Specimen data is less susceptible to errors in taxonomy than observation records due to the existence of a specimen that can have its identification verified by tradition determination [49] or through genetic sequence data [24]. Observation records are accumulated with lower effort, albeit with less certain verification [17]. Closer links between these record types could benefit the application of biodiversity data in ecological research. However, our results show that both observation and specimen records are susceptible to taxonomic, spatial, temporal and environmental biases, and that these biases differ between record types. These differences need to be accounted for in order to optimally link specimen and observation data.

We found that specimen and observation records differed in the temporal bias displayed. Specimens tended to be older, with the majority of observation records dating from the most recent 15 years, clearly supporting our hypothesis (H1). This reflects advances in the availability of electronic biological recording systems [17]. Since there are other differences in bias between these data types (e.g. in space, environment, see below), studies incorporating a temporal element (for example investigating shifts in species distribution over time) will be susceptible to the differential biases between the record types [50]. Specimen records were also sampled on average three days later than preserved specimens. This may reflect ambitions to collect mature specimens for herbaria collections. The magnitude of this difference is similar to the decadal advance in flowering phenology of 4.5 days of British plants [51]. Differences between record types should be therefore accounted for within phenological studies that use occurrence data (e.g. [52]).

Different taxa showed different patterns of bias across specimen and observation records. As hypothesised (H2), there was a greater diversity in the specimen record than the observation record, and there were more specimen records than observation records for most species. This highlights the importance of natural history collections as archives of biodiversity [30]. Furthermore, there was greater evenness between species in terms of number of records in the specimen record than observations (although it should be noted that the species abundance curve shows low evenness for specimens too). The most commonly sampled species where not the same in each record type. In the observation record type the most recorded species, *Lupinus polyphyllus*, is a common invasive plant species. The species most recorded as observations are widespread and abundant flowering plants, including trees, shrubs, grasses and herbs; all of which are easy to identify. In contrast, most of the ten most collected species as specimens are graminoids. These species are often difficult to identify under field conditions. In addition, many of the most collected species have been the subject of taxonomic disagreements within the Norwegian plant systematics (e.g. [53]), and hence calls for the collection and deposition within herbaria of specimens for further study.

The number of occurrence records increased with species geographic range for both data types. The relationship was steeper and with greater variance for observations, suggesting that the representation of more common species within natural history collections becomes saturated at lower levels than observations; this supports our third hypothesis. The most widespread and recorded species included a number of woody plants. These are very common species within Norway but are not well represented in herbarium collections, partly due to the challenges in preserving woody plants. A number of species were notable for being outliers in the relationship between geographic range and number of occurrence records (Fig 3). These include a number of invasive plant species such as *Lupinus polyphyllus* and *Solidago canadensis*, represented on the Norwegian Black List, an ecological risk assessment of alien species in Norway [54]. The high abundance of occurrence records of these species is therefore likely to have been driven by a drive to assess the spread of these invasive species.

The species richness data should be interpreted with caution. The number of species recorded in total is for the main phyla in this study greater than the known number of species in Norway [55]. This deviation highlights potential errors within the data set. These errors may be in taxonomy (e.g. misidentification) or errors in georeferencing. Alternatively, these species may be observations of recently introduced species or species only occurring in domestic or horticultural settings. However, the differential results in terms of taxonomic composition and species richness found between the record types demonstrate that biodiversity studies should strongly emphasise specimen data over observation records.

The most prevalent species recorders contributed a large proportion of the total data set, with the maximum contributions exceeding 5% of the total species occurrences in this data set. There was a longer tail of recorders who had collected one or two specimens, while fewer recorders contributed to the species observations record, in opposition to our hypothesis (H4). Our estimate of the number of records made by each recorder is likely to be conservative due to alternative formatting of recorders names as well as co-authored records. The spatial and temporal distribution of specimen records within natural history collections has been linked to the operating pattern of local experts [29, 56]. On the basis of our findings, we assert that even in a large data set (with almost 5 million records) the distribution of both observation and specimen records is, to a large extent, driven by idiosyncrasies in the operating localities of a small number of prolific biodiversity recorders.

Spatial biases in sampling of biodiversity data often affect ecological inference [7, 47], unless accounted for. Biases vary between different data sources and even between different natural history collections [6]. Records are often more concentrated in more accessible localities, for example near to roads [57]. In our study we found different relative geographical biases between occurrence record types. In contrast to our hypotheses (H5), observations were more aggregated than specimens. Crucially, there was very low concordance between the record types in terms of spatial distribution. Non-vascular plants showed greater spatial bias in specimen records than observation records. This was driven by greater observation of these phyla along the western coast of Norway, compared to greater collection of preserved specimens around Oslo, Trondheim and the Kongsvoll area, Dovrefjell. Part of the latter may be related to lags in data digitalization of herbaria data as the large bryophyte herbaria in Oslo and Bergen (estimated over 200 000 specimen records) are not yet databased and published. For all phyla, both observation and specimen data were recorded more often in regions of Norway with high population density than expected. This likely highlights the key role of accessibility in determining the distribution of species occurrence data [57, 58].

The spatial difference in distribution of Bryophyta and Marchantiophyta occurrence records translated into differences in sampled climatic space. Observation records were more frequent in regions with higher and more seasonal precipitation than specimen records. Both observation and specimens of all three phyla were overrepresented in warmer regions of Norway and regions with higher human population density; these patterns support our hypothesis (H6). The differential biases in environmental conditions sampled may impact on ecological inferences made from these data. Such biases have clear implications for understanding species distributions and ecology (e.g. [29]). There are further concerns regarding the lack of concordance between the record types. For example, distribution ecology for some taxa may be better known from some regions, while the evolutionary or physiological ecology (both of which require specimens) may be better understood within different regions with contrasting environmental conditions. However, understanding the nature of this difference, as facilitated by this study, will allow for the benefits of both species-occurrence data types to be better utilised within ecological and conservation applications.

### Conclusions and recommendations

Species occurrence data exists both as specimens held in natural history collections and observations for which there is no physical specimen; taxonomic errors are presumed to be less frequent in the specimen record. There is thus potential for further research to develop methodologies to facilitate the validation of observation records using specimens records. Our study demonstrates that observations and specimens have different biases in time, space, taxonomic coverage and environment. These differential biases should be accounted for when assessing the quality of species occurrence data. For the use of species occurrence data, one solution is to include observation records only if they fall inside the known species range as validated by specimen collections (e.g. [24]).

Since natural history collections are under more direct management than species observations, we here make recommendations to natural history collections to increase synergies between the record types by structuring collection of specimens, and documenting collecting strategies. This would allow verification of the less structured observation data [59]. We recommend that natural history collections make further efforts to manage their collecting activities in order to better link to the increasing availability of observation data by: 1. Common and widespread species should be better represented in collections. 2. Taxa should be collected from throughout the taxon’s geographic range. 3. Density of collections of taxa should be stratified to reflect the geographic distribution of species observations of each taxon. These steps will allow better exploitation of the complementary advantages of observation and specimen species occurrence records within ecological and evolutionary research and conservation.

## Supporting information

S1 Table The most prevalent recorders of species occurrences as human observations or preserved specimens. (DOCX)

S1 Fig The proportion of plant occurrences within each record type found within 10 x 10 km cells across Norway. (DOCX)

S2 Fig Pairwise correlations between all phyla and record types. (DOCX)
