# Supplementary material for: Contrasting spatial, temporal and environmental patterns in observation and specimen based species occurrence data
Source: PLoS One. 2018 Apr 26;13(4):e0196417. doi: 10.1371/journal.pone.0196417 (PMC5919666; doi:10.1371/journal.pone.0196417)
Supplement: S1 Table — (DOCX) [file pone.0196417.s001.docx]

**S1 Table**. **The most prevalent recorders of species occurrences as human observations or preserved specimens** (other than unknown recorders). The proportion of the total number of occurrences within each record type made by each recorder is shown in parentheses. Note that names were used as supplied so this (nor Figure 2b) does not take into account differences in name format (e.g. Joe Bloggs; Bloggs, Joe; J. Bloggs, nor co-recorded occurrences e.g. Joe Bloggs and Jane Doe).

| Rank | Human observation | Number of records (proportion) | Preserved specimens | Number of records (proportion) |
| --- | --- | --- | --- | --- |
| 1 | Lid, Johannes | 247 459 (0.067) | Tore Ouren | 21 906 (0.020) |
| 2 | Wischmann, F. | 177 894 (0.048) | Finn Wischmann | 21 035 (0.020) |
| 3 | Pedersen, Oddvar | 120 798 (0.033) | R. E. Fridtz | 17 527 (0.016) |
| 4 | Steinar Stueflotten | 104 659 (0.028) | Reidar Elven | 16 388 (0.015) |
| 5 | Jordal, John Bjarne | 102 015 (0.028) | Eli Fremstad | 16 091 (0.015) |
| 6 | Anders Breili | 99 044 (0.027) | Ove Dahl | 14 412 (0.013) |
| 7 | Wischmann, Finn | 59 786 (0.016) | R. Tambs Lyche | 13 596 (0.013) |
| 8 | Øystein Folden | 58 269 (0.016) | Trond Skoglund | 12 584 (0.012) |
| 9 | Kaasa, Jon | 47 936 (0.013) | Johannes Lid | 11 570 (0.011) |
| 10 | Holten, Jarle I. | 40 755 (0.011) | Per Størmer | 11 451 (0.011) |
