# Supplementary material for: Contrasting spatial, temporal and environmental patterns in observation and specimen based species occurrence data
Source: PLoS One. 2018 Apr 26;13(4):e0196417. doi: 10.1371/journal.pone.0196417 (PMC5919666; doi:10.1371/journal.pone.0196417)
Supplement: S1 Fig — (DOCX) [file pone.0196417.s002.docx]

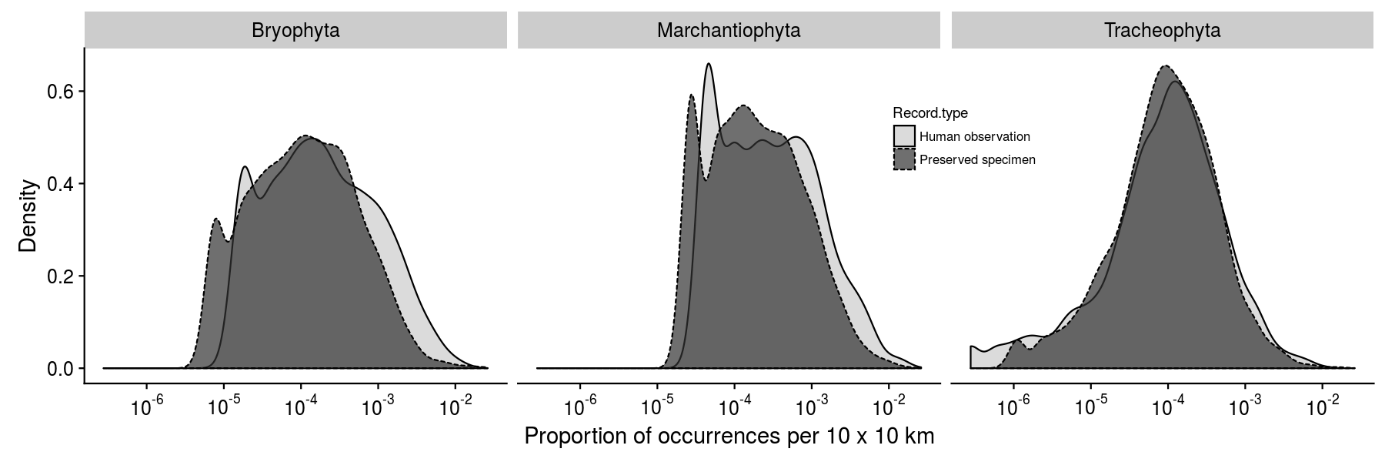


**S1 Fig**. **The proportion of plant occurrences within each record type found within 10 x 10 km cells across Norway**. Data are shown for each individual phyla. Note log_10_ x axis. Distributions towards the right denote phyla for which the record-type base is more concentrated in few cells.
