# Supplementary material for: Contrasting spatial, temporal and environmental patterns in observation and specimen based species occurrence data
Source: PLoS One. 2018 Apr 26;13(4):e0196417. doi: 10.1371/journal.pone.0196417 (PMC5919666; doi:10.1371/journal.pone.0196417)
Supplement: S2 Fig — (DOCX) [file pone.0196417.s003.docx]

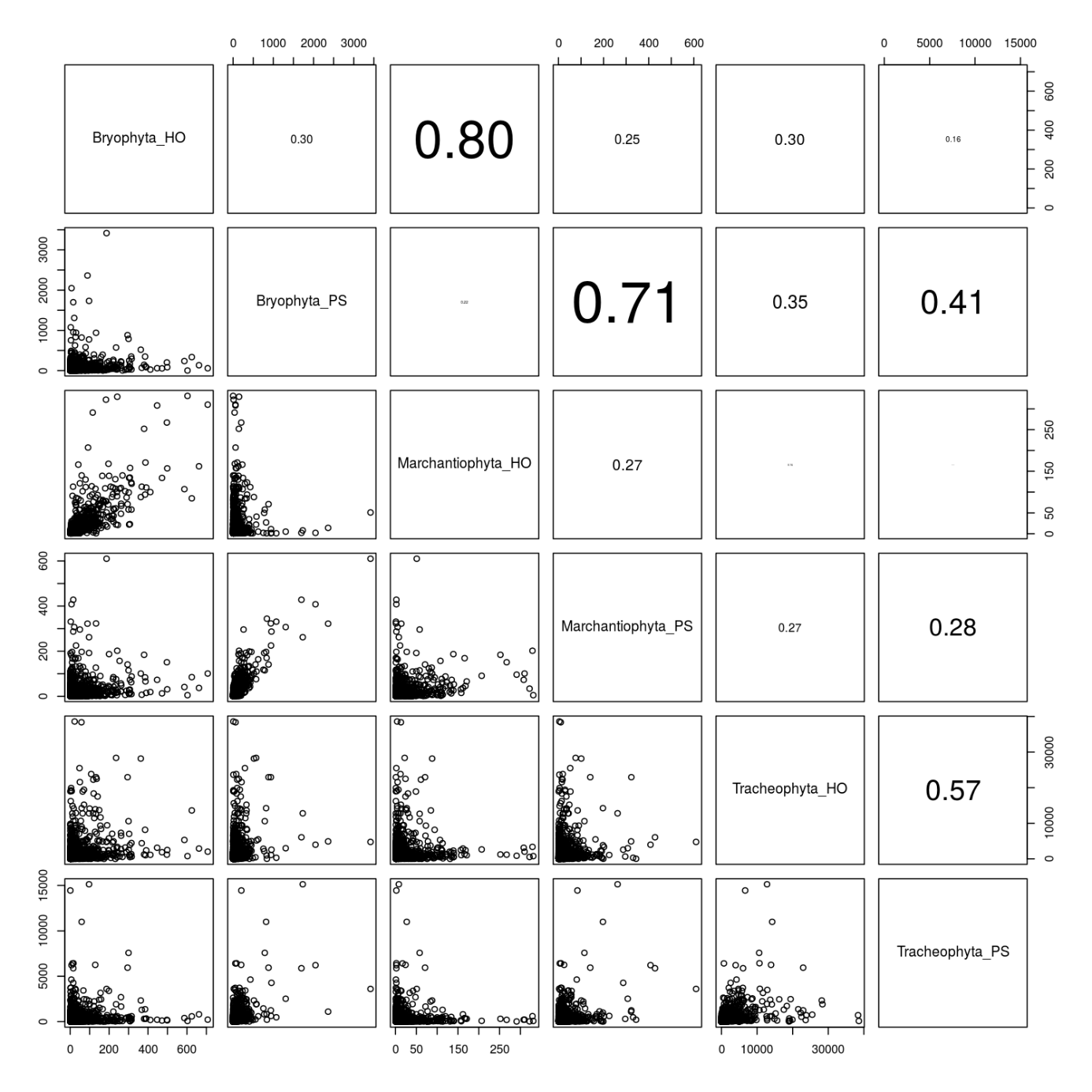


**S2 Fig**. **Pairwise correlations between all phyla and record types**. HO denotes human observation and PS preserved specimens. Each data point is a 10 x 10 km cell plotted along axes of the number of records in that cell (or human population density). Lower panels show pairwise plots, and upper panels show Spearman rank correlation coefficients, with the text size being proportional to the absolute value.
